# Cross-Cultural Adaptation and Validation of the Listening Inventory for Education-Revised in Italian

**DOI:** 10.3390/audiolres14050069

**Published:** 2024-09-14

**Authors:** Maria Nicastri, Hilal Dincer D’Alessandro, Karen Anderson, Miriana Ciferri, Luca Cavalcanti, Antonio Greco, Ilaria Giallini, Ginevra Portanova, Patrizia Mancini

**Affiliations:** 1 Department of Sense Organs, Sapienza University of Rome, 00185 Rome, Italy; maria.nicastri@uniroma1.it (M.N.);; 2 Department of Audiology, Faculty of Health Sciences, Istanbul University-Cerrahpaşa, 34500 Istanbul, Turkey; 3 Supporting Success for Children with Hearing Loss, Tampa, FL 33625, USA

**Keywords:** children, hearing loss, classroom performance, acoustics, cochlear implants, LIFE-R, mainstream education

## Abstract

Background: Listening difficulties may frequently occur in school settings, but so far there were no tools to identify them for both hearing and hearing-impaired Italian students. This study performed cross-cultural adaptation and validation of the Listening Inventory for Education-Revised for Italian students (LIFE-R-ITA). Methods: The study procedure followed the stages suggested by the Guidelines for the Process of Cross-cultural Adaptation of Self-Report Measures. For the content validation, six cochlear implanted students (8–18 years old) pre-tested the initial version. Whenever any situation did not occur in Italy, the item was adapted to more typical listening situations in Italy. The final version of LIFE-R-ITA was administered to a sample of 223 hearing students from different school settings and educational degrees in order to collect normative data. Results: For the LIFE-R-ITA, hearing students showed an average score of 72.26% (SD = 11.93), reflecting some listening difficulties. The subscales (LIFE total, LIFE class, and LIFE social) indicated good internal consistency. All items were shown to be relevant. Most challenging situations happened when listening in large rooms, especially when other students made noise. LIFE social scores were significantly worse than those of LIFE class (*p* < 0.001). Conclusions: The present study provides cross-cultural adaptation and validation for the LIFE-R-ITA along with the normative data useful to interpret the results of students with hearing loss. The LIFE-R-ITA may support teachers and clinicians in assessing students’ self-perception of listening at school. Such understanding may help students overcome their listening difficulties, by planning and selecting the most effective strategies among classroom interventions.

## 1. Introduction

School represents the place where children and adolescents spend most of their daily time, participating in learning activities under the guidance of their teachers whilst sharing meaningful interactions and social experiences with their peers. Throughout the school day, they have to attend lectures, follow instructions to accomplish learning tasks, participate in classroom debates, understand questions during oral exams, and communicate during collaborative group activities or social interactions. More than 50% of these experiences are driven by verbal messages [1] that require good speech perception [2]. The presence of noise in classrooms and other school environments may negatively impact students’ understanding of spoken messages, learning, and social participation [2]. Indeed, the presence of noise considerably deteriorates speech perception [3] and significantly associates with an indiscriminate filtering out of noise [4], leading to the engagement of cognitive resources and an increased listening effort to maintain successful communication [5]. When this demand is prolonged over time, listening fatigue occurs. Schoolchildren who report more listening difficulties in their classrooms show greater fatigue, and a greater risk of annoyance [6] and stress [7], than those who report fewer listening difficulties in their classrooms [8].

Communicating and learning in challenging listening conditions at school result in well-documented negative effects on various cognitive functions, such as attention [9], memory [10], concentration [11], problem-solving abilities [12,13], and creativity [14]. Moreover, high levels of noise seem to reduce motivation and negatively contribute to dysfunctional student behaviors [13]. In the long term, constant and repeated noise exposure at school potentially impacts overall academic success, reducing students’ performances in reading, mathematics, and spelling abilities [15,16,17,18].

Consequently, noise pollution and attention, which are important aspects of understanding spoken language, become even more compelling for students, who are not yet adult-level listeners. In fact, the development of a mature language continues until approximately 15 years of age [19,20], and individuals younger than 15 years have less linguistic knowledge and listening experience. Therefore, they may rely less on the redundancy of speech signals to fill in missing words or phrases whose clarity is reduced by competing signals in noisy environments [19,21]. For example, typically hearing children in the first and third grades show poorer speech-in-noise perception than adults [22].

If noisy environments show significant negative effects on students with typical hearing, the presence of background noise and/or reverberation might be even more challenging for students who are deaf or hard of hearing (DHH). Indeed, they may have more difficulty in understanding speech in noisy listening conditions, which require greater auditory attention, and this fact may lead to greater fatigue and cognitive load [23]. This additional energy spent on hearing, processing, and understanding speech may lead to some physical symptoms such as headache and may have psychological consequences such as annoyance, which refers to a series of feelings including discomfort, sadness, disappointment, and frustration [24]. Noise annoyance may result in irritability, nervousness, concentration difficulties, and decreased motivation, and may negatively affect DHH children’s speech understanding, language comprehension, vocabulary learning, and academic achievements [25,26,27,28,29].

Considering all these aspects, in 2002, the American National Standards Institute (ANSI), along with the Acoustical Society of America and the U.S. Access Board, established the first classroom standard (ANSI S12.60-2002) [30], known as the Acoustical Performance Criteria, Design Requirements, and Guidelines for Schools Standard. They recommended that the level of noise in unoccupied classrooms up to 20,000 cubic feet in size should not exceed 35 dBA and the reverberation time should be no longer than 0.6 s [30]. These requirements have been adopted by the World Health Organization guidelines on community noise to allow for good quality teaching and learning conditions [31]. To this end, the American Speech-Language-Hearing Association National Standard on Classroom Acoustics suggests that the signal-to-noise ratio (SNR) in classrooms should be at least 15 dB [32]. The SNR should be even more favorable for younger students so that they can perform at the same level as older children [21,33], e.g., at least 20 dB to allow for good speech perception in DHH children when compared with hearing peers under identical noisy listening conditions [34]. Lower noise levels in school environments have been shown to be significantly correlated with lower levels of stress and higher levels of participation in school activities, creativity, and a positive educational experience [35,36].

Despite the knowledge of the detrimental effects of noise and published guidelines on noise management, schools are unlikely to meet these standards. A recent study by Gremp et al. [37] measured the sound levels of the background noise in 38 classrooms in Canada. The findings indicated that none of the classrooms, whether general education or separate instruction, met the general ANSI standard of 35 dB for unoccupied classrooms. The ANSI recommendation was even stricter for unoccupied classrooms where DHH students attended lectures; however, the same problem was observed. Measured levels ranged from 37.2 to 66.2 dB with means from 43.5 to 53.5 dB for unoccupied classrooms and reached means from 62.6 to 65.1 with a range from 44.2 to 75.3 when students were present and language arts instruction was taking place. Similar results were found by Wang and Brill [38], who logged sound levels acquired across six days in 220 classrooms and reported an average level of noise of 64 dBA (SD = 2.5), ranging from 58 to 73 dBA. The average SNR between non speech and speech sounds was 16.9 dBA, which is within the ASHA limit for hearing children. However, 27.3% of the classrooms did not meet the minimum SNR, whereas 84.1% exhibited average SNRs below 20 dBA, failing to achieve the SNR recommended for DHH students. Results from other studies were even more critical, reporting SNRs ranging from 5 to 7 dB [39].

Perceptive difficulties in school environments may depend on many factors such as internal/external noise, acoustic characteristics of various school settings (i.e., gym, classroom, school canteen), class size, student/teacher position, and type of classroom activities (i.e., individual learning, group work, traditional teaching, class discussions). Hence, any attempt to improve listening at school requires collecting information about real school characteristics along with personal listening experiences and difficulties. For this purpose, in 1999, Anderson and Smaldino [40] developed the Listening Inventory for Education (LIFE), which could be used by clinicians and teachers to collect information about students’ perceptions of the acoustic environment at school and their listening experience in the classroom. Such a tool intended to allow for identification of personal listening needs and monitoring of the effects of solutions and changes they implement to meet these needs. The revised version of the tool (LIFE-R), available since 2012, is designed for students starting from the third grade until they complete high school education [41]. It is a self-report instrument in which individual students rate their own perceptions of the listening environment and quality of hearing in various classrooms, schools, or social situations. This tool consists of three sections. The first section is called “Before LIFE”. This section consists of six multiple-choice questions to query students’ general perceptions of how well they hear in the classroom, where they typically sit in relation to the teacher, the noise they hear, and how they feel about listening with their hearing technology. The second section of the LIFE-R is the “Student Appraisal of Listening Difficulty”. This section intends to help students to provide more detailed information about their listening environment, proposing and describing 15 typical classroom, school, or social listening scenarios. Scenarios 1–10 (LIFE class) focus on classroom listening situations, and Scenarios 11–15 (LIFE social) focus on school or social listening situations. For each listening scenario, students have to indicate their hearing quality on an 11-point Likert scale. This scale allows respondents to assign a numerical value, which is also associated with a descriptor, to indicate their rating for each listening scenario: 10 points (always easy), 7 points (mostly easy), 5 points (sometimes difficult), 2 points (mostly difficult), or 0 points (always difficult). Finally, the third section, called “After LIFE” consists of six multiple-choice questions to explore the strategies that the students use and the actions that they take when faced with challenging listening situations. Among the various response options provided by the questionnaire, they can select as many options as apply to them. Therefore, total n represents the total number of responses received (or actions taken) for each category, but not the number of respondents. The English version was first released in paper-and-pencil format, but since 2012 it has been made in a free, online format, available on the Teacher Tools Takeout website (http://teachertoolstakeout.com (accessed on 18 June 2024). item 0100). A new version of the online form has been developed in 2023 (https://lifer.successforkidswithhearingloss.com/ (accessed on 18 June 2024). Interested people can download the questionnaire and complete it online, contributing to the collection of broad information about the functional classroom listening experiences of DHH students. The questionnaire is also available in other languages such as Hebrew, Arabic, Welsh, and Dutch [42].

To date, there has been a lack of tools to identify listening difficulties in school settings for both hearing and DHH students in Italy. The aim of the present study was to realize cross-cultural adaptation and validation of the LIFE-R for Italian students.

## 2. Materials and Methods

Before starting the present study, consent for the Italian adaptation of the LIFE-R was obtained from the authors [41]. Cross-cultural adaptation followed the stages suggested by the Guidelines for the Process of Cross-cultural Adaptation of Self-Report Measures [43]. The reliability and validity procedures were in accordance with the Consensus-Based Standards for the Selection of Health Status Measurement Instruments (COSMIN) checklist [44]. This study was conducted in accordance with the ethical requirements of the 1964 Declaration of Helsinki, its later amendments, and the existing legislation in Italy. The present protocol was approved by the local ethics committee of Sapienza University of Rome (Protocol no: 259/2020). Informed consent was obtained from the study participants or their parents.

### 2.1. Cross-Cultural Adaptation

Following the Authors’ consent, the first stage of the adaptation was forward translation. Forward translations (T1 and T2) of the tool from English to Italian were performed by two independent translators with Italian as the mother tongue. The first translator was a speech and language therapist with audiological knowledge (MN), whereas the second translator did not have any specific audiological background (a professional translator not included in the list of the authors).

The translators, independently, wrote a report on their own translations with comments on challenging words/phrases or uncertainties, which were later discussed and resolved. Stage II involved the synthesis of the forward translations, made in the presence of both translators and an audiologist as a recording observer (PM). A written report from Stage II was used to address the issues and form a consensus. A new written report carefully documenting the synthesis process was produced and a synthesized translation (T-12) of the tool was developed. In Stage III, the T-12 version of the tool was translated back into the original language by two independent translators with English as the mother tongue (professional translators not included in the list of the authors). Both were blinded to the original version of the LIFE-R and had no relevant medical background. As soon as back translations (BT1 and BT2) were available, all the materials were checked by an expert committee, composed of two ENT specialists (AG, LC), a speech therapist (MC), an audiologist (HDD), a methodologist (an external statistician), and a linguist (an external professional) in addition to all the translators. The role of the expert committee was to consolidate all versions of the questionnaire and to develop a pre-final version for field testing. Therefore, the committee reviewed all translations and reached a consensus on any discrepancy.

### 2.2. Content Validation

Following the example of Krijger et al. [42] for the Dutch version of the LIFE-R, content validation of the LIFE-R-ITA was performed in a small sample of six DHH students, recruited at the Cochlear Implant Center of the Sapienza University of Rome. The participants were 8–18 years old and enrolled in mainstream elementary (n = 2), middle (n = 2), or high school education (n =2). All patients were cochlear implants users. The questions were validated through semi-structured interviews using a probe technique. After each question, the following probe question was asked: “Che cosa significa?” (What does this mean?). This technique enabled us to verify whether the translation had been properly interpreted and validated.

### 2.3. Concept Validation and Cross-Cultural Adaptation

Each item of the tool was checked for equivalence, considering listening situations in Italy as opposed to typical American conditions. DHH students were asked whether each specific listening situation identified in the tool also occurred in their school or classroom. Whenever a situation did not occur in Italy, with the consent of authors, the item was adapted to more typical listening situations in Italy. The final version is presented in Appendix S1.

### 2.4. Participants and Normative Data

The study consisted of six schools located in different regions of Italy (two schools each from the Southern, Central, and Northern regions). Informed consent was shared with the schools, which helped recruit students. Informed consent was obtained from the parents of students aged <18 years and directly from students aged ≥18 years. To facilitate data collection, the final version of the LIFE-R-ITA was converted to an online version using the EUSurvey system. A total of 223 typically hearing participants met the recommended sample size, ranging from two to 20 participants per item to reduce the risk of errors when studying reliability [45].

In Italy, the following grades exist: elementary school (five grades from 5–6 to 10 years of age), middle school (three grades from 11 to 13 years), and high school (five further grades from 14 to 18–19 years). The educational settings are presented in Table 1.

Similar to Krijger et al. [42], questions related to hearing loss and technology were omitted from the online version used to collect data from typically hearing study participants. Furthermore, question 6 was omitted from both before and after LIFE-R-ITA (‘How do you feel about listening with your hearing equipment in the classroom’ and ‘What would you do if your listening technology is not working?’). Omitted answers were reported as ‘OM’ in the analyses.

In the Italian version, the question regarding the fish bank was removed from the before LIFE-R since no classroom in Italy had it inside.

### 2.5. Statistical Analysis

The construct validity, internal consistency, and cross-cultural validity of the scale were assessed by following the Consensus-Based Standards for the Selection of Health Status Measurement Instrument (COSMIN) checklist [44]. The construct validity of the scale was calculated through exploratory factor analysis for the LIFE class and LIFE social, and the Kaiser–Meyer–Olkin value was calculated; Bartlett’s sphericity test was performed. Cronbach’s alpha was used to examine the internal consistency of the LIFE-R-ITA. Alpha values of 0.7, 0.8, and 0.9 represented a fair, good, and excellent degree of internal consistency, respectively [46]. Cronbach’s alpha was calculated separately for 15 listening situations (LIFE total), the class (LIFE class), and social listening situations (LIFE social).

Differences between groups and classes were determined using the parametric Student’s *t*-test and/or the non-parametric Wilcoxon, Mann–Whitney or Kruskal–Wallis tests depending on the normality of the data, which were evaluated using QQ plots and Shapiro–Wilk tests. *p* values less than or equal to 0.05 were considered statistically significant for all statistical analyses. IBM^®^ SPSS^®^ (Chicago, IL, USA) was used to perform statistical analysis.

## 3. Results

### 3.1. Translation and Linguistic Validation

The best versions of the items or their combinations were chosen during the meeting for the synthesized version of the forward translations. The syntactic structure of the sentences was kept simple, considering the minimum age of students to whom the tool was destined (8 years of age). As there were some issues regarding some words that needed to be changed to have a single version for all school grades, the authors were contacted to ask for their approval (see Appendix S2 for agreed cross-cultural changes in the questionnaire).

During the meeting between the translators and the expert committee for the assessment of the equivalence between the backward translation and the original version, the material at the disposal of the expert committee included the original questionnaire, all the translations (T1, T2, T12, BT1, and BT2), and the reports written about them. Decisions were made regarding the semantic, idiomatic, experiential, and conceptual equivalence between the original English LIFE-R and the Italian version. All ambiguities were resolved on the basis of feedback from the authors of the original version and our multidisciplinary committee. Thus, the LIFE-R-ITA version was finalized (Appendix S1).

### 3.2. Content Validation

None of the six DHH students had problems interpreting the questions and possible answers. However, in some cases, they needed assistance with complex sentence structures. Therefore, in the final version, complex sentences from verbatim translation were replaced with semantically equivalent sentences to facilitate reading and comprehension.

### 3.3. Concept Validation and Cross-Cultural Adaptation

All but one listening situations, one item of the “Student Appraisal of Listening Difficulty”, described in the LIFE-R-ITA were typical for Italian school settings as well. Pre-tested DHH students reported that the listening situation of announcements in the classroom (item 12: announcements) did not occur in Italy as there are no loudspeakers for announcements in Italian schools. This item was replaced by the item ‘How well do you understand the teacher in gym class?’ and renamed as “listening in a gym class”.

Furthermore, an addition was made to the item 13 (large classroom) to make it suitable for all Italian school grades. Indeed, school meetings and assemblies are situations that occur regularly in Italian secondary schools but not in elementary schools. With the consent of the authors, we added “school play”, which is typical of the Christmas/Easter period and the last school day just before the summer vacation in elementary and middle schools. Such events are organized in school theatres, large classrooms, or school halls (depending on the available space of the school) where several classes of students are involved in both rehearsals and final performance. Thus, item 13 became “There is a school play, a school meeting or an assembly. Many classes of students are sitting together. Students are listening to a teacher. The teacher is speaking without a microphone. How well can you hear and understand the words the teacher is saying?”.

### 3.4. Weighting Scores

Similar to Krijger et al. [42], for the Italian version of the LIFE-R- Student Appraisal of Listening Difficulty section, the total number of items remained identical to the original version, allowing us to maintain the original scoring method. For each item, the scores ranged from 10 (always easy) to 0 (always difficult). Three scores were obtained:

- LIFE total: the sum score of all items, for a maximum score of 150
- LIFE class: the sum score of all items, for a maximum score of 100
- LIFE social: the sum score of all items, for a maximum score of 50

Each score can also be expressed as a percentage or as an average of the scores for each item. The latter was used in this study.

### 3.5. Normative Data

A total of 223 students (124 females and 99 males) were recruited to collect normative data for the LIFE-R-ITA. Their school grades varied from elementary third to high school fifth grade. Their ages ranged from 8.15 to 18.61 years. The mean number of students in each class ranged from 20.69 (SD = 3.26) to 24.36 (SD = 2.9). There were no statistically significant differences in class size across school grades (H = 0.32, *p* = 0.57). Details are reported in Table 1.

### 3.6. Before LIFE-R-ITA

**Figure 1:**
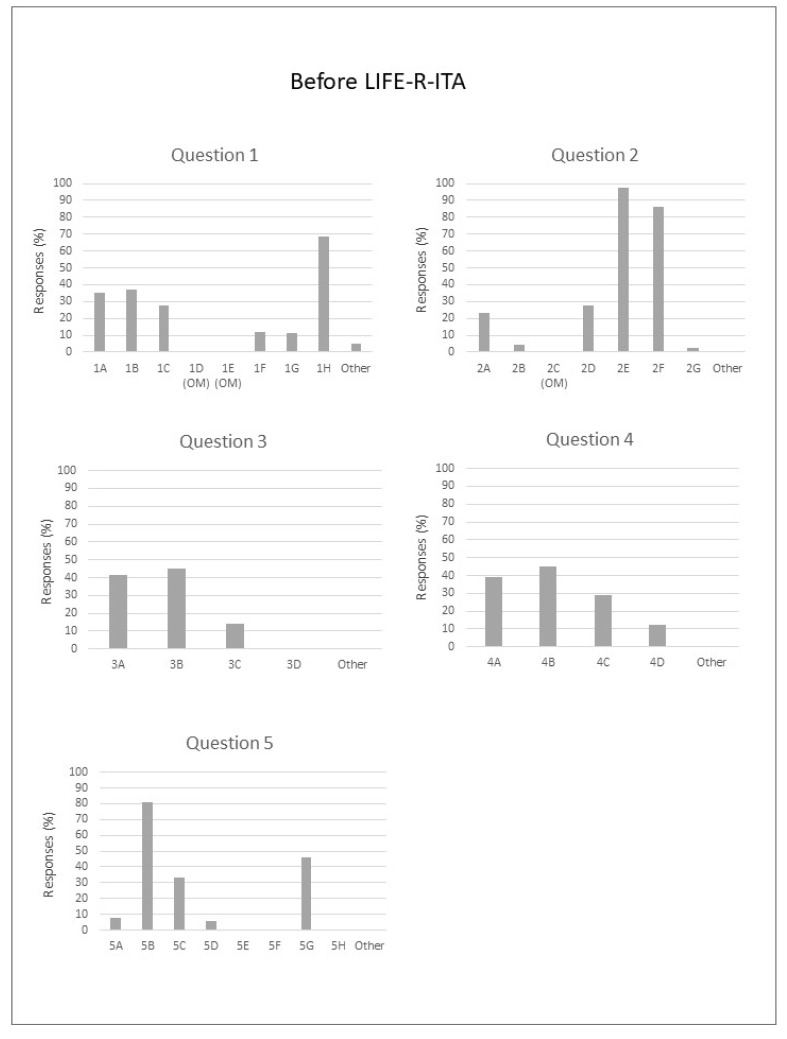
Percentage of responses for each Before LIFE-R-ITA item.

Figure 1 shows the results of the multiple-choice questions from before LIFE-R-ITA. Bar graphs represent the percentage of cases indicated by the students, who were allowed to select multiple answers for each question. Omitted answers are indicated by the letters “OM”. Students in the sample were similarly distributed across desks in the classroom (questions 1A, B, and C), and about 68% indicated that their position was close to where the teacher stood to talk to the class (question 1H).

The main sources of noise in the classroom were the noise of other students inside (99%) and/or outside (91%) the classroom (questions 2D and E).

Most of the students stated that their teachers were easy to hear, and they could hear almost everything the teacher said (questions 3A and B), whilst ‘not at all’ and ‘other’ options were not selected at all (questions 3C and D). Hearing students indicated that most teachers spoke from the same location or moved around for a short period (questions 4A and B), whilst ~30% of them taught moving around for about half or more than half of the time. Finally, for question 5 (How do you know when you did not hear or understand the teacher completely?), almost all hearing students asked for help by asking the teacher or classmates to repeat what has been said (questions 5B and G).

### 3.7. Student Appraisal of Listening Difficulty LIFE-R-ITA

To evaluate the construct validity of the Student Appraisal of Listening Difficulty LIFE-R-ITA section, the exploratory factor analysis (EFA) was used separately for the classroom (LIFE class) and social situations (LIFE social).

For classroom situations (items 1–10), the Kaiser–Meyer–Olkin coefficient was 0.823, and Bartlett’s test of sphericity was statistically significant (*p* < 0.001). The EFA maintained all 10 items that explained 63.94% of the variance, which was contained in the two major factors. Factor 1 included the first seven items, whereas Factor 2 included the remaining three items (Table 2).

Cronbach’s alpha for the classroom situations was 0.835, reflecting good internal consistency. All items were shown to be relevant and contribute to the questionnaire, given that if any item was eliminated, the alpha value decreased, consequently decreasing internal consistency.

For social situations (items 11–15), the Kaiser–Meyer–Olkin coefficient was 0.822, and Bartlett’s test of sphericity was statistically significant (*p* < 0.001). The EFA maintained all five items that explained 62.16% of the variance, which was contained in the two major factors (Table 3).

Cronbach’s alpha for social situations was 0.701, demonstrating good internal consistency. All items were shown to be relevant and contribute to the questionnaire, given that if any item was eliminated, the alpha value decreased, consequently decreasing internal consistency.

**Figure 2:**
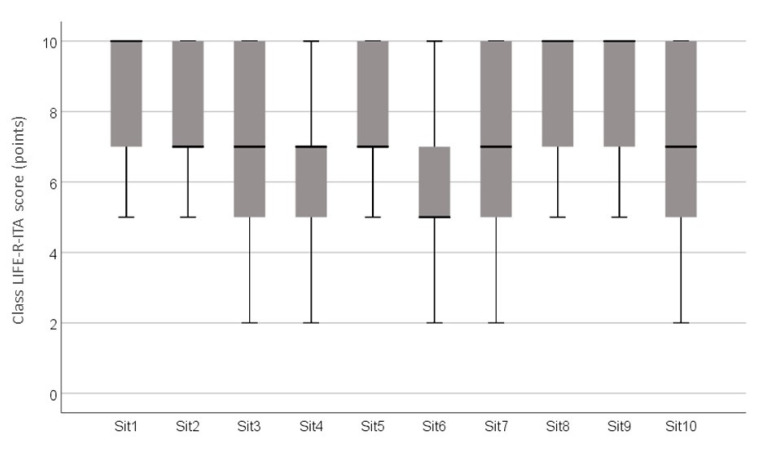
Mean, minimum, and maximum scores for each item of the Student Appraisal of Listening Difficulty LIFE-R-ITA. Class section.

**Figure 3:**
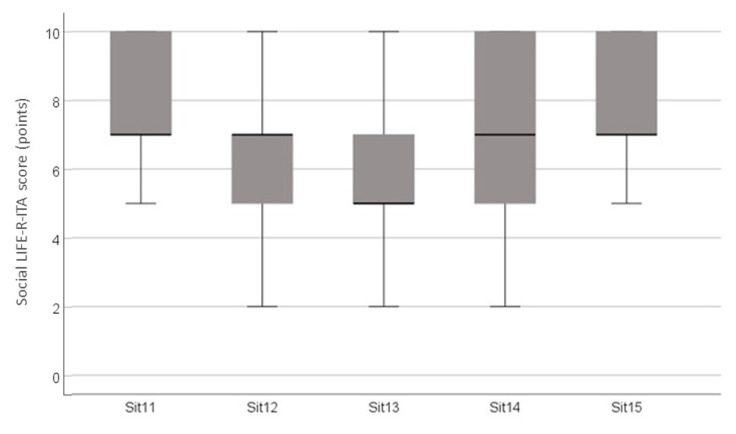
Mean, minimum, and maximum scores for each item of the Student Appraisal of Listening Difficulty LIFE-R-ITA. Social section.

The score for the Student Appraisal of Listening Difficulty LIFE-R-ITA section was, on average, 72.26% (SD = 11.93%), for a total of 15 LIFE listening situations (Figure 2 and Figure 3). Most challenging situations were listening in a large room (Sit13, 5.7 ± 2.14), listening when other students make noise (Sit6, 6.06 ± 2.2), listening during a gym class (Sit12, 6.07 ± 1.72), and students answering during a discussion (Sit4, 6.5± 2.2). Social listening situations (LIFE social) were experienced by hearing students as more effortful than classroom listening situations (LIFE class): students reported 7.5 (SD = 1.28) vs. 6.7 (SD = 1.34), respectively, and the differences were statistically significant (Student’s *t* = 10.51, *p* < 0.001).

The LIFE class scores were significantly worse for middle school students (Kruskal–Wallis H = 12.36, *p* = 0.002): Middle school students showed an average score of 7.05 (SD = 0.97), whilst the corresponding scores were 7.75 (SD = 1.3) and 7.72 (SD = 1.19) for the elementary and high school students, respectively. No statistically significant gender differences were detected in the TOTAL scores (Mann–Whitney U = 6068, *p* = 0.88). The LIFE class scores were significantly correlated with class size (Spearman’s rho = −0.76, *p* < 0.001).

### 3.8. After LIFE-R-ITA

**Figure 4:**
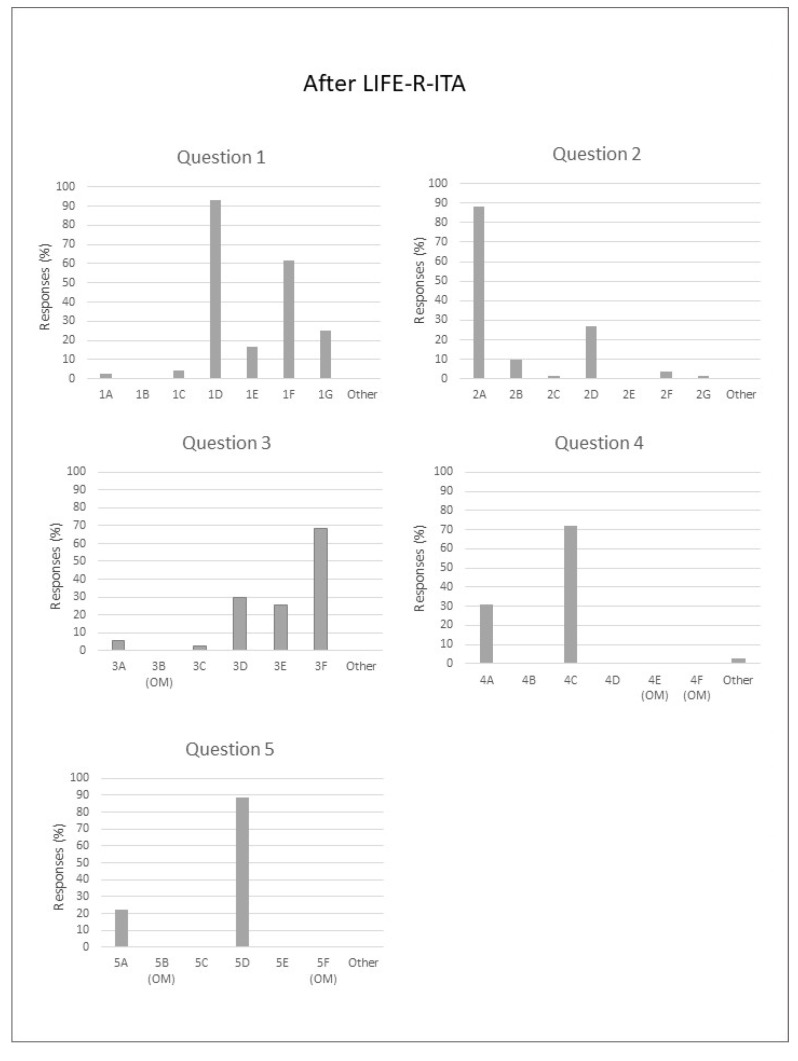
Percentage of responses for each After LIFE-R-ITA item.

Figure 4 shows After LIFE-R-ITA responses to all the five questions. In the classroom, most students used assertive strategies to indicate to the teacher that they could not hear or comprehend what the other students said (questions 1D–F), asking them for more information or to repeat what was said. About 10% also used the strategy of asking the teacher after class (question 1G). More than 80% of students reported their listening difficulties to their teachers when there was too much noise in the classroom (question 2A), but in almost a quarter of the situations, they did not report their problem and tried to listen more carefully (question 2D), whilst 10% simply looked around and glared at people making noise, hoping the teacher would notice. Assertiveness also involved reporting the problem to a teacher very softly (70% of the sample verbally reported the problem to the teacher); ~30% turned around in their seat or moved, trying to see the student’s face more easily, whilst ~25% of them showed a passive behavior, doing nothing.

In social situations, students prevalently reported missing only parts of the messages, so they could ask for them to be filled in (~70%). Only 30% asked to move to a quieter place or stay closer to the speaker to hear and see better. Finally, regarding the question of communicating in noisy places, students preferred, as the prevalent strategy, to attempt to stop the noise or to move away from it (question 5D), and ~20% tried to avoid noisy places when they were expected to listen and talk (question 5A).

## 4. Discussion

The presence of noise in classrooms often becomes inevitable, owing to limitations in the architectural/acoustic characteristics of schools (e.g., the location of the school, lack of acoustic isolation, reverberation, and classroom size). This fact represents an important question that needs special attention to minimize listening challenges at schools as well as to provide optimal learning conditions for students. Poor acoustic conditions reduce speech intelligibility and interrupt communication between teachers and students [47], and negatively affect students’ speech perception [48], attention [49], response time [48], reading ability [50], memory [51], and general wellbeing [52], thus negatively impacting academic achievements. Not surprisingly, such significant effects become even more critical for hearing-impaired students, who are shown to be more sensitive to the presence of noise [39].

The availability of assessment tools to help identify typical listening situations and listening challenges at school is, therefore, important to select strategies that may help minimize listening difficulties. Since no behavioral assessment tools for this purpose are available in Italian, the present study aimed to perform a cross-cultural adaptation and validation of the LIFE-R developed by Anderson et al. [41]. The procedure involved forward/backward translation and reconciliation, which required various steps so that the Italian version could explore target behaviors similarly to the original version. Some minor adaptations were needed to allow for the tool to cover all listening situations that a student might face in Italian schools (e.g., school plays) as well as to avoid those that are not typical (e.g., fish tank in the classroom or announcements from the loudspeakers) without changing the nature and number of items of the original version. For this cross-cultural adaptation, special effort was made to keep the level of language as simple as possible. A group of DHH students, recruited at the Cochlear Implant Centre of Sapienza University of Rome to assess the content validity, confirmed that the Italian adaptation was clear and comprehensible. They could respond to all items without needing any support (e.g., photos), suggesting that the tool can be used with students having a minimum age of 8 years.

Exploratory factor analysis allowed us to show that the tool has good content validity: Bartlett’s test of sphericity indicated that the correlation matrix was not random, whilst the Kaiser–Meyer–Olkin measure of sampling adequacy was 0.823 for the LIFE class and 0.822 for the LIFE social, reflecting adequate sampling [53]. It also showed that there was no overlap between the items, all of which were critical for the total explained variance. For the LIFE class, 10 items were retained in two components, whilst the LIFE social comprised one component, all with eigenvalues above 1 (Kaiser et al., 1960) [54]. The explained variance was greater than 60% for both sections, further strengthening the construct validity of the tool [55].

Normative data were collected from a sample of 223 students, covering an age range from 8 to 19 years, balanced across three Italian school grades (the last three classes of elementary school as well as middle and high schools). Statistical analysis showed that the LIFE-R-ITA had good internal consistency for both the LIFE class and LIFE social sections. Further studies in diverse samples of different regions, socioeconomic backgrounds, and educational settings may further strengthen the validity of the LIFE-R-ITA.

In the before LIFE-R-ITA section, hearing students reported that, in almost 70% of the cases, they were located close to where the teacher stands to talk to the class, reflecting the attention of teachers to stay in positions that allow for better listening conditions to their students. The most frequent noise sources were the students themselves, both inside and outside the classroom. This was followed by in-class equipment such as fans or computers. Such findings are in line with students’ reports from the Krijger et al. [42] study in Belgium. Likewise, a recent study conducted in Italy supported such subjective reports through physical measurements of seven types of typical school scenarios in three different Italian cities (Florence, Perugia, and Rome). For a total of 29 schools, the authors reported that multimedia interactive whiteboards, projectors, fans, and air conditioners were the most frequent noise sources in unoccupied classrooms. In occupied classrooms, students’ noise differed by approximately 4–7 dBA during interactive activities (group activities, audio/video supported interactive activities, etc.) compared to traditional lecturing [56].

Similarly to the study by Krijger et al. [42], students also reported that their teachers speak predominantly from the same place almost all the time whilst walking around for just a short time. In about 40% of the cases, the teachers also moved more frequently, at least 50% of the time. A teacher’s position in the classroom and proximity to students may have significant effects on student motivation, engagement, behavior, and self-efficacy [57]. Hence, teachers can change their position in the classroom to engage students according to different aspects such as type of lessons, whether they are more or less interactive, specific activities performed by students, student behaviors, and/or physical characteristics of the classroom (size, desk positions, technical equipment disposition, etc.).

The total score on the Student Appraisal of Listening Difficulty LIFE-R-ITA was 72.26%, confirming the experience of listening difficulties in typically hearing Italian students. Such findings were consistent with the study by Krijger et al. [42]. The level of difficulty varied across different classrooms, schools, or social settings. The most difficult listening situations seemed to happen when students were listening to a person speaking without any microphone in a large room or at a school assembly, or when they were participating in a gym class where several students made noise. All of these situations are very common in school settings and the level of noise typically exceeds national recommendations [58]. For example, in a study investigating level of noise in high schools’ gymnasiums, Carvalho and Barreira [59] found that background noise level ranged from 30 to 59 dB LAeq in the absence of physical education classes and from 68 to 90 dB LAeq during physical education lessons, a together with long reverberation time at 0.5–1–2 kHz (from 2.5 to 8.1 s). Further difficulties in listening to the teacher when other classmates are talking or when noise levels are high compared to when the classroom is quiet have also been reported in other studies [52,60,61]. This is an important aspect considering that speech levels exceeded 65 dBA on average 35% of the day, and non-speech levels exceeded 50 dBA on average 32% of the day. Speech levels exceeding 65 dBA are statistically more common in the third and eighth grades [38]. This fact results in poor SNRs, often ranging from +5 to −7 dB SNR [39], rather than the +10- and +15-dB SNR that are recommended for optimal hearing and learning in the classroom [19]. Moreover, in large rooms, background noise is accompanied by other important aspects such as distance and reverberation. Distance affects the speech level whilst reverberation significantly deteriorates its quality, smoothing formant transitions by imposing on temporal and spectral cues of the speech signal [21,62]. If noise, distance, and reverberation, even alone, show significant effects on speech understanding, their combination may result in greater detrimental effects, reducing the likelihood that students can effectively communicate in school settings (in the classroom or outside) [19].

Similarly to Krijger et al. [42], statistically significant differences were observed between classroom and social listening in the Italian student sample. The LIFE social analyses situations such as listening at gym classes, assemblies, and common areas, characterized by higher levels of noise than the classrooms [56,59], making verbal communication more challenging and leading to fatigue, boredom, loss of concentration, and physical symptoms such as headache [56].

Unlike the Krijger et al. study [42], which showed a trend toward greater listening difficulties at higher school grades, in the present Italian sample, scores from elementary and high school students were similar, whereas significantly worse scores were reported by subjects in middle school. Such findings might be linked to the challenging transition period from elementary to middle school. This challenge involves a change in the school environment that occurs during significant cognitive and physiological changes of puberty [63]. Respecting elementary school children is a common teacher behavior in Italy. However, school transition requires significant cognitive and behavioral adaptation as it often involves not only a change in physical location but also changes in the educational perspective and instructional approach, increases in the number of teachers, subjective decreases in teacher support, increases in class size, changes in peer networks, and greater expectations of personal responsibility [64]. Such challenges are accompanied by increasing demands on the child’s skills in development and might be at the basis of students’ perception of greater difficulty in listening and understanding.

Previous studies have reported that the transition from middle to high school is even more chaotic and problematic, with a significant increase in workload [65]. Moreover, teachers are sometimes perceived as cold, impersonal, and insensitive to students’ needs [66,67]. The lack of such reflections in Italian students’ self-reports might be due to several reasons. Students coming from middle school are already used to interacting with more teachers, so they may have already learned how to cope with this aspect. Although adolescence is a period of rapid physical, psychological, and social developmental changes that may be considered stormy in the early stages, adolescents achieve numerous skills with age, enabling them to manage complex abstract thinking, improve behavioral awareness, management, and control, as well as to develop personal identity and greater autonomy in the middle and late stages [68]. The sum of these abilities may help them use effective strategies to better manage challenging listening situations and enable students in high school to have a more positive perception of listening at school. Consistent with this suggestion, a study by Minichilli et al. [69] found significantly higher levels of environmental noise annoyance among lower secondary school students (11–13 years old) than among upper secondary ones (14–18 years old). Another explanation might be the differences in linguistic competences between students in middle and high schools. Given that the maturation of language skills continues until about 15 years of age [19,20], younger subjects have less linguistic knowledge and listening experience. Therefore, they may rely less on the redundancy of speech signals to fill in missing words or phrases whose clarity is reduced by competing signals in noisy environments [19,21]. Consequently, younger students might be less positive about their listening experiences than older students.

Finally, in the present sample, the number of students in the classroom was similar across grades. The better scores in high schools compared to middle schools might be due to differences in other aspects that affect noise exposure in schools, such as the location and structural characteristics of the building, and/or classroom exposition and acoustics [56].

Regarding possible gender differences, no statistically significant differences in the total scores were observed between the males and females, in accordance with other studies investigating the self-assessment of noise annoyance during daily life at school [69].

The total score was also inversely related to the number of students in the classroom, and this relationship was strong. Such results were consistent with those of Alqahtani et al. [70] who investigated various factors that may significantly influence classroom noise. Likewise, the authors found that class size was one of the most significant factors, along with the use of technological devices and the duration of lectures.

The after LIFE-R-ITA allowed to record the students’ degree of assertiveness to resolve their listening difficulties in both classroom and in social contexts. Assertiveness is an important skill that enables individuals to avoid extreme behaviors such as aggressiveness and complete subjection [71], as well as to express their emotions, defend their goals, and establish interpersonal relationships in a positive, adaptive, and healthy way [72]. Most students in all three grades reported using assertive strategies, such as asking the teachers for more information or repeating what they said, as well as specifically signaling the problem (e.g., a student whose voice is too quiet). However, approximately 20–30% of students use more passive behaviors, such as simply looking around or trying to listen more carefully without signaling the problem. Further studies in DHH students are needed to effectively compare hearing and DHH students as well as to capture listening difficulties across different groups. The tool may help identify these students and support them in gaining awareness of their difficulties, reasoning critically and independently about their difficulties, and then acting assertively to communicate and solve problems [73]. Moreover, implementing longitudinal studies may help assess how well the LIFE-R-ITA tracks changes in listening difficulties over time and its effectiveness in guiding interventions.

## 5. Conclusions

Questionnaires are among the most commonly used tools for assessing both students’ and teachers’ noise perception, annoyance, disturbance, listening difficulties, and speech comprehension. In Italian, there was a lack of tools to identify listening difficulties in school settings for both hearing and DHH students. The LIFE-R-ITA, a validated cross-cultural adaptation of the original English LIFE-R, may support teachers and clinicians in assessing students’ self-perception of listening in the classroom and other typical listening situations at school. Data were collected from a sample of 223 hearing students from different school settings and educational degrees and can be used as a reference when assessing DHH students’ listening difficulties. Identifying self-perceptions of listening difficulties represents the first step in gaining insight into possible solutions to help students overcome these difficulties, by planning and selecting the most effective strategies among classroom interventions such as the use of assistive listening devices or other technological tools as well as by strengthening student self-advocacy skills. The tool may also allow us to verify the effectiveness of problem-solving strategies, and, if not effective, to modify them in a positive cycle aimed at reducing listening barriers and realizing the best possible learning opportunities for every student.

## Figures and Tables

**Table 1:** Number and characteristics of students for each school grade.

	Students Number	Mean Chronological Age (SD)	Mean Class Size (SD)
**Elementary**			
**3**	26	8.55 (0.26)	20.69 (3.26)
**4**	21	9.53 (0.37)	21.62 (2.29)
**5**	27	10.34 (0.25)	22.15 (2.66)
**Middle School**			
**1**	21	11.8 (0.56)	22.9 (21.1)
**2**	19	12.36 (0.22)	23.79 (1.75)
**3**	33	13.2 (0,48)	23.3 (1.94)
**High School**			
**1**	18	14.4 (0.25)	23.5 (2.53)
**2**	18	15.46 (0.69)	22.89 (2.17)
**3**	11	16.6 (0.31)	22.55 (2.88)
**4**	15	17.02 (0.24)	21.73 (2.76)
**5**	14	18.12 (0.37)	24.36 (2.9)

**Table 2:** Construct validity—exploratory factor analysis (EFA) for LIFE-R-ITA class.

Items	Factor 1	Factor 2
**1 Teacher in front**	0.643	
**2 Teacher with back turned**	0.884	
**3 Teacher moving**	0.600	
**4 Student answering during discussions**	0.661	
**5 Directions understanding**	0.703	
**6 Students making noise in class**	0.620	
**7 Noise outside the classroom**	0.751	
**8 Multimedia**		0.692
**9 Listening with fan noise**		0.844
**10 Large and small group simultaneously**		0.804
**Eigen values 4.08 1.32**		
**Variance explained 47.77 16.17**		
**Total variance explained 63.94**		

**Table 3:** Construct validity—exploratory factor analysis (EFA) for LIFE-R-ITA social.

Items	Factor 1
11 Cooperative small group	0.754
12 Gym class	0.648
13 Large room	0.773
14 Outside	0.611
15 Informal social times	0.794
Eigen value 2.36	
Variance explained 62.16	

## Data Availability

The raw data supporting the conclusions of this article are available in https://data.mendeley.com/datasets/d2msxmb52v/1 (accessed on 11 September 2024).

## Supplementary Materials

The following supporting information can be downloaded at: https://www.mdpi.com/article/10.3390/audiolres14050069/s1, Appendix S1 Listening Inventory For Education-Revised-Italian (LIFE-R-ITA); Appendix S2: Cross-cultural changes in the questionnaire.
