# Supplementary material for: Cross-Cultural Adaptation and Validation of the Listening Inventory for Education-Revised in Italian
Source: Audiol Res. 2024 Sep 14;14(5):822–39. doi: 10.3390/audiolres14050069 (PMC11417904; doi:10.3390/audiolres14050069)
Supplement: Supplementary file 1 [file audiolres-14-00069-s001.zip › Appendix S1.pdf]

Appendix S1

Listening Inventory For Education-Revised-Italian (LIFE-R-ITA)

Nome____ **Anno**_______**Data** ____________________________

Insegnante_______________________________**Classe**_______________ **Scuola**_________________________________

□ Apparecchio acustico **□ Impianto cocleare** **Tipo di tecnologia uditiva usata in classe**____________________

Periodo di prova □No □Si Durata_____________ **Risposte al LIFE-R considerando le prestazioni □con □senza uso di sistema FM**

Before LIFE-R-ITA: *Domande per gli studenti*

Fra tutte quelle indicate, segna tutte le situazioni che si verificano in classe. Se ti capita di trovarti anche in situazioni diverse oltre a quelle elencate, puoi aggiungerle e descriverle alla voce "altro".

1. **Segna la/le risposte che descrivono meglio la tua posizione all'interno della classe:**
   - O Il mio posto è nella prima o seconda fila dell'aula.
   - O Il mio posto è nella fila centrale dell'aula.
   - O Il mio posto è nell'ultima fila dell'aula.
   - O Quando sono al mio posto, l'orecchio da cui sento meglio è rivolto verso l'insegnante che parla.
   - O Quando sono al mio posto, l'orecchio da cui sento peggio è rivolto verso l'insegnante che parla.
   - O Il mio posto è vicino a una fonte di rumore all'interno della classe.
   - O Il mio posto è vicino a una fonte di rumore proveniente dall'esterno della classe.
   - O Il mio posto è vicino a dove si trova l'insegnante per parlare alla classe.
   - O Altro:____

2. **Quali suoni (rumori) senti quando sei in classe? (Tali suoni possono essere presenti anche solo occasionalmente)**
   - O Rumore di ventole all'interno dell'aula (es. ventilatore).
   - O Rumore proveniente dal sistema di riscaldamento/raffreddamento interno l'aula (es. condizionatore).
   - O Rumore dei computer all'interno dell'aula.
   - O Rumore prodotto dagli studenti all'interno dell'aula.
   - O Rumore prodotto da studenti fuori dalla tua classe (es. dal corridoio o dal cortile della scuola).

- O Suoni dalle lampade fluorescenti (es. luci al neon).
- O Altro:____

3. Quando sei seduto al tuo posto abituale in classe, quanto senti bene l'insegnante quando parla?

- O Molto bene, l'insegnante è facile da ascoltare.
- O Bene, posso ascoltare e capire quasi tutto.
- O Non molto bene, perdo diverse cose di ciò che dice.
- O Male, perdo la maggior parte delle cose che dice l'insegnante.

4. Qual è la migliore descrizione della posizione del tuo insegnante in classe durante le lezioni?

- O Parla fermo nello stesso posto quasi tutto il tempo.
- O Parla camminando un po' per la classe solo ogni tanto, forse una volta o due al giorno.
- O Parla la metà del tempo da diversi posti nella classe.
- O Parla quasi sempre spostandosi in diversi posti nella classe.

5. Cosa fai quando non hai sentito o capito bene l'insegnante?

- O Ho difficoltà a iniziare il mio lavoro perché non capisco cosa l'insegnante vuole che io faccia.
- O So che dovrei chiedere all'insegnante di ripetere ciò che è stato detto.
- O Mi guardo intorno per vedere cosa stanno facendo gli altri studenti.
- O Seguo le istruzioni dell'insegnante in modo sbagliato.
- O Guardo le labbra dell'insegnante per capire ciò che dice.
- O Rispondo in modo inappropriato o non rispondo.
- O Chiedo ad un compagno di ripetermi cosa ha detto l'insegnante.
- O Non so quando non ho sentito o non ho capito l'insegnante.
- O Altro:____

6. Come ti senti quando puoi ascoltare in classe usando _________________________(inserire il nome del sistema di supporto all'ascolto in uso)?

- O Sono contentissimo perché riesco a sentire e capire meglio.
- O Mi sento nervosa.
- O Sono imbarazzato e ho vergogna.
- O Sono contento.
- O Non ne ho idea
- O Altro:____

Listening Inventory For Education-Revised-Italian (LIFE-R-ITA)

di Karen L. Anderson, PhD, Joseph J. Smaldino, PhD, & Carrie Spangler, AuD

Nome____ **Anno**_______**Data** ____________________________

Insegnante_______________________________**Classe**_______________ **Scuola**_________________________________

□ **Apparecchio acustico** □ **Impianto cocleare** **Tipo di tecnologia uditiva usata in classe**____________________

Periodo di prova □**No** □**Si** **Durata**_____________ **Risposte al LIFE-R considerando le prestazioni** □**con** □**senza uso di sistema FM/streaming**

Valutazione difficoltà d'ascolto- Studente

Istruzioni: Cerchia la risposta che descrive meglio il tuo livello di difficoltà d'ascolto in ognuna delle situazioni descritte.

LIFE-R-ITA Situazioni di ascolto in classe		E' Sempre Facile	Per lo più È Facile	A volte è difficile	Per lo più è difficile	E' sempre difficile
**Domande**	**Breve descrizione**					
1. L'insegnante sta parlando di fronte alla classe. I compagni di classe sono in silenzio. Ognuno sta guardando e ascoltando l'insegnante. Quanto bene riesci a sentire e capire le parole che dice l'insegnante?	1. L'insegnante parla di fronte all'aula	**10**	**7**	**5**	**2**	**0**
2. L'insegnante sta parlando ma ti dà le spalle mentre scrive alla lavagna o guarda in faccia un altro studente. Tu non riesci a vedere il volto dell'insegnante. Quanto bene riesci a sentire e capire le parole che dice l'insegnante quando non puoi vedere il suo volto?	2. L'insegnante parla di spalle	**10**	**7**	**5**	**2**	**0**
3. L'insegnante sta parlando. Lei/lui sta anche camminando, muovendosi intorno all'aula. Quanto bene riesci a sentire e capire le parole che dice l'insegnante se non puoi vedere il suo volto ed è dall'altra parte della stanza?	3. L'insegnante parla mentre si muove	**10**	**7**	**5**	**2**	**0**
4.A volte gli insegnanti fanno delle domande durante una lezione. Un compagno di classe che è seduto dall'altra parte dell'aula rispetto a te sta rispondendo alla domanda. Quanto bene riesci a sentire e capire le parole che sta dicendo lo studente? √*Condizione tipica:* □ *Con microfono FM/streaming usato dallo studente* □*Senza microfono FM/streaming*	4. Studente che risponde durante una discussione	**10**	**7**	**5**	**2**	**0**
5. L'insegnante sta spiegando come dovresti svolgere un compito. Dà indicazioni solo una o due volte. Quanto bene riesci a sentire le parole e a capire cosa l'insegnante vuole che tu faccia?	5. Comprensione delle istruzioni	**10**	**7**	**5**	**2**	**0**
6. L'insegnante sta parlando. Alcuni compagni di classe fanno rumore ai loro posti. Potrebbero cercare dei fogli, far cadere matite, sussurrare o muovere i piedi. Quanto bene riesci a sentire e capire le parole che dice l'insegnate mentre i compagni fanno rumore?	6. Altri studenti fanno rumore	**10**	**7**	**5**	**2**	**0**
7. L'insegnante sta parlando. Tu senti del rumore fuori dalla classe. Potrebbero essere studenti nel corridoio, il cortile fuori, voci dalla porta accanto, macchine o aeroplani. Quanto bene riesci a sentire e capire le parole che sta dicendo l'insegnante?	7. Rumore fuori dall'aula	**10**	**7**	**5**	**2**	**0**
8. Tutti stanno guardando il computer, la TV o uno schermo video. L'insegnante sta mostrando un video o stai ascoltando qualcosa mostrato sullo schermo del computer. Quanto bene riesci a sentire e capire le parole dette mentre guardi lo schermo?	8. Multimedia (video, computer)	**10**	**7**	**5**	**2**	**0**

9. L'insegnante sta parlando alla classe. L'insegnante sta usando un proiettore che fa rumore oppure l'aria fuoriesce dal condizionatore nella tua aula. Quanto bene riesci a sentire e capire le parole dell'insegnante quando c'è un rumore simile ad una ventola allo stesso tempo?	9. Ascolto con rumore di ventole	**10**	**7**	**5**	**2**	**0**
10.Un insegnante sta parlando davanti alla classe. Un altro insegnante sta parlando contemporaneamente ad un piccolo gruppo di studenti. Quanto bene riesci a sentire e capire le parole che sta dicendo l'insegnante davanti?	10. Grande e piccolo gruppo contemporaneamente	**10**	**7**	**5**	**2**	**0**
**Somma elementi 1-10 (Max 100)** **PUNTEGGIO DI ASCOLTO IN CLASSE Pretest score__________. Post-test score__________.** Data: Data:						

Copyright 2011 di Karen L. Anderson, PhD, Joseph J. Smaldino, PhD, & Carrie Spangler, AuD

**L.I.F.E Situazioni di ascolto aggiuntive/sociali a scuola**						
**Domande**	**Breve descrizione**	**Sempre facile**	**Per lo più è facile**	**A volte è difficile**	**Per lo più è difficile**	**Sempre difficile**
11. I compagni di classe lavorano tutti in piccoli gruppi. Ogni gruppo sta parlando e spostando dei fogli. Quanto bene riesci a sentire e capire le parole che stanno dicendo i compagni del tuo gruppo?	11. Apprendimento cooperando in piccoli gruppi	**10**	**7**	**5**	**2**	**0**
12. Tu, i tuoi compagni e l'insegnante siete in palestra. L'insegnante vi parla e dà istruzioni sulle attività da svolgere. Quanto bene riesci a sentire e a capire le parole dette dall'insegnante in palestra?	12. Palestra	**10**	**7**	**5**	**2**	**0**
13. C'è una riunione di classe o un'assemblea. Sono sedute insieme molte classi di studenti. Gli studenti stanno ascoltando un'insegnate. L'insegnate parla senza microfono. Quanto bene riesci a sentire le parole dette dall'insegnante?	13. Ascoltare in una stanza grande	**10**	**7**	**5**	**2**	**0**
14. Sei fuori con altri studenti che conosci. Può essere una lezione di ginnastica all'aperto o siete insieme nel cortile, alla fermata dell'autobus o in gita. Qualcuno parla mentre degli studenti fanno rumore. Quanto bene riesci a sentire e capire quando le persone parlano all'aperto?	14. Ascoltare gli altri all'aperto	**10**	**7**	**5**	**2**	**0**
15. Sei a scuola, esci con alcuni studenti che conosci. Potrebbe essere l'ora di pranzo il momento di togliere i cappotti, o di andare verso la classe. C'è rumore. Quanto chiaramente riesci a sentire e a comprendere cosa dicono gli altri studenti?	15. Ascoltare gli studenti durante il tempo ricreativo	**10**	**7**	**5**	**2**	**0**
**Somma degli elementi 11-15 (Max 50) PUNTEGGIO DI ASCOLTO IN SITUAZIONI AGGIUNTIVE Pretest score__________. Post-test score_________.** Date: Date:						

_ _

Delle 15 domande di Listening Inventory For Education, le seguenti situazioni sono state valutate come

Sempre difficile (✿✿✿), Per lo più difficile (✿✿), o A volte difficile (✿):

Per:

Data:

Da:

✿	______________________ principali condizioni di ascolto difficile
	1. L'insegnante parla di fronte alla classe
	2. L'insegnante parla di spalle
	3. L'insegnante parla in movimento
	4. Studente che risponde durante una discussione
	5. Ascoltare e comprendere istruzioni
	6. Altri studenti fanno rumore
	7. Rumori fuori dall'aula
	8. Multimedia (video, computer)
	9. Ascoltare con rumore di ventole attive
	10. Grande e piccolo gruppo contemporaneamente
	11. Apprendimento cooperativo in piccoli gruppi
	12. Palestra
	13.Ascoltare in una grande stanza
	14. Ascoltare gli altri quando si è fuori
	15. Ascoltare gli studenti durante il tempo ricreativo i momenti di socialità non formali
Più ✿ quanto più è difficile. Nessuna ✿ = nessun problema.	

10 Situazioni di ascolto in classe
Punteggio percentuale =

%

5 Situazioni di ascolto aggiuntive
Punteggio percentuale =

%

Copyright 2011 di Karen L. Anderson, PhD, Joseph J. Smaldino, PhD, & Carrie Spangler, AuD

Listening Inventory For Education-Revised-Italian (LIFE-R-ITA)

di Karen L. Anderson, PhD, Joseph J. Smaldino, PhD, & Carrie Spangler, AuD

Nome____ **Anno**_______**Data** ____________________________

Insegnante_____________________________**Classe**______________ **Scuola**________________________________

□ Apparecchio acustico **□ Impianto cocleare** **Tipo di tecnologia uditiva usata in classe**____________________

Periodo di prova **□No** **□Si** **Durata**____________ **Risposte al LIFE-R considerando le prestazioni □con □senza uso di sistema FM**

After LIFE-R-ITA: *Domande per gli studenti*

Rispondi a queste domande scegliendo le risposte che descrivono quello che fai tu nella situazione indicata (fai una crocetta sulla lettera; puoi segnare anche più di una risposta). Se hai una risposta non indicata, aggiungila alla riga "altro".

1. Che cosa fai per far capire al tuo insegnante che non hai sentito o capito ciò che ha detto?

a) Uso un'espressione facciale per fargli sapere che mi sono perso alcune informazioni (ad esempio l'espressione di chi è perplesso).
b) Uso uno specifico segnale che il mio insegnante e io abbiamo concordato (come ad esempio mettermi il dito sul mento).
c) Non faccio nulla e spero di trovare il modo di capirlo più tardi.
d) Alzo la mano e chiedo maggiori informazioni (come ad esempio "Cosa dobbiamo fare a pag 300?"; "Facciamo i problemi con le divisioni?).
e) Mi guardo intorno per vedere cosa stanno facendo gli altri studenti (sperando che l'insegnante si accorga che guardo in giro).
f) Alzo la mano e chiedo all'insegnante di ripetere ciò che ha detto.
g) Chiedo spiegazioni all'insegnante alla fine della lezione.
h) Altro:____

2. Cosa fai se in classe c'è troppo rumore e fai fatica a capire l'insegnante?

a) Alzo la mano per comunicare al mio insegnante che sto avendo difficoltà a capire a causa del rumore.
b) Mi guardo intorno e lancio un'occhiataccia (con una faccia contrariata) alle persone che stanno facendo rumore (sperando che l'insegnante se ne accorga).
c) Mi alzo e chiudo la porta (se il rumore è nel corridoio) o cambio posto, mettendomi lontano dal rumore.
o) Non faccio nulla, mi concentro di più nell'ascolto e spero di sentire abbastanza per capire cosa stia dicendo l'insegnante.
e) Mi alzo dal mio posto e con tranquillità chiedo alla persona che fa rumore di smettere (il mio insegnante sa che questa è una delle mie strategie di ascolto).

f) Inizio a fare qualcos'altro perché l'insegnante è troppo difficile da ascoltare e capire (sperando che l'insegnante si accorgerà che non sono attento).
g) Parlo con l'insegnante alla fine della lezione.
e) Altro: ____

3. Cosa fai quando durante una discussione di gruppo in classe un tuo compagno parla con la voce troppo bassa e non riesci a capire cosa dice?
a) Non faccio nulla e spero che il mio compagno non stia dicendo cose importanti.
b) Alzo la mano e ricordo all'insegnante di passare il microfono FM/streaming al compagno prima che lui inizi a parlare alla classe.
c) Guardo da un'altra parte rispetto al compagno che sta parlando, sperando che il mio insegnante lo noterà e si ricorderà che per me è difficile ascoltare le voci basse.
d) Mi guardo intorno o mi sposto in modo da poter vedere più facilmente la faccia dello studente che sta parlando.
e) Non faccio nulla e spero che ciò che il compagno sta dicendo venga poi ripetuto dall'insegnante.
f) Alzo la mano e dico qualcosa del tipo "Maria ha una voce bassa e non ho sentito tutto ciò che ha detto".
g) Altro: ____

4. Che cosa fai quando non riesci a sentire o capire quello che dicono i tuoi amici quando state uscendo?
a) Chiedo di spostarci in un posto più tranquillo o mi avvicino alla persona che sta parlando così posso sentire meglio e vedere la sua faccia.
b) Non faccio niente e spero solo che non mi facciano domande perché a volte le mie risposte non sono corrette e loro ridono di me.
c) Di solito perdo soltanto piccole parti del messaggio, quindi dico qualcosa come "Qual era il nome del film?" o "Chi si è trovato nei guai?"
d) A volte inizio a parlare di qualcos'altro, così loro mi ripeteranno l'argomento e io saprò di cosa stiamo parlando.
e) I miei amici sanno che a volte ho difficoltà a sentire tutto. Quando mi perdo qualcosa, tocco il braccio di chi sta parlando e faccio una faccia interrogativa così si ricorda di guardarmi in faccia.
f) Comincio a guardarmi intorno e a volte quando faccio questo loro si ricordano di guardarmi in faccia quando parlano.
g) A volte mi allontano perché è troppo difficile seguire la conversazione.
e) Altro: ____

5. Cosa fai quando cerchi di comunicare e c'è rumore?
a) Cerco di evitare luoghi dove c'è rumore.
b) Uso il sistema FM e passo il microfono a chi sta parlando.
c) Smetto di prestare attenzione - se le persone vogliono che capisca qualcosa, me lo diranno di nuovo.
d) Cerco di eliminare il rumore o di allontanarmi dal rumore e di avvicinarmi alla persona che parla.
e) Non faccio nulla e spero che nessuno mi chieda nulla.

f) Cambio programma sul mio ausilio uditivo (protesi acustica o impianto cocleare) e metto quello impostato per "ambiente rumoroso".
g) Altro: ____

6. Cosa fai se il tuo ausilio uditivo non funziona?
a) Informo subito il mio insegnante alzando la mano o usando un segnale concordato con lui.
b) Mi siedo al mio banco senza dire nulla e spero che riprenda a funzionare.
c) Lo dico al mio insegnante alla fine della giornata scolastica.
d) Sostituisco le batterie e cerco di capire perché non funziona (es. provo a cambiare il cavetto).
e) Dico al mio insegnante che c'è un problema e poi vado dalla persona che di solito mi aiuta quando ci sono. dei problemi con le protesi acustiche o l’impianto cocleare o il sistema FM/streaming.
f) Altro: ____
