# Supplementary material for: Cross-Cultural Adaptation and Validation of the Listening Inventory for Education-Revised in Italian
Source: Audiol Res. 2024 Sep 14;14(5):822–39. doi: 10.3390/audiolres14050069 (PMC11417904; doi:10.3390/audiolres14050069)
Supplement: Supplementary file 1 [file audiolres-14-00069-s001.zip › Appendix S2.pdf]

Appendix S2:

Listening inventory for education – revised (L.I.F.E.-R.) – student version

TRANSLATION CHANGES APPLIED AFTER THE CHECK OF THE BACK TRANSLATIONS

General linguistic changes

- "Kids": kids in English could be used to refer both children and boys/girl; in Italian there is not a word with both the meaning as we have bambini (children) and ragazzi (for people 11-18 years old); so to avoid the need for two version of LIFE-R, one for children 8-10 years old and the other for subjects 11-18 years old, we used the word "students".
- "Face": in Italian can be translated as "viso", "Faccia", "volto"; we decided to use "Volto"
- "Play ground": the literal translation would be "parco giochi" but in Italy the school have not a play ground, unless they are nursery or pre-schools; In general Italian schools have "cortili" (courts)
- "Papers": "fogli" is the more correct Italian translation than "carte", both possible

Specific changes within the items

ITEM 1 → No linguistic inconsistencies or conceptual errors were detected so no changes were performed;

ITEM 2 → after checking the two back translations , we decided to change "di fronte ad" with "guarda in faccia", a translation closer to the English verb form "to face";

ITEM 3 → after checking the two back translations, the term "nell'aula" has been corrected as "intorno" because it is more faithful to the concept expressed in English ("around of");

ITEM 4 → after checking the two back translations, the term "FM – Mic" has been reinserted;

ITEM 5 → No linguistic inconsistencies or conceptual errors were detected so no changes were performed;

ITEM 6 → No linguistic inconsistencies or conceptual errors were detected so no changes were performed;

ITEM 7 → after checking the two back translations, the singular form "noise" was corrected as "... del rumore" to respect the singular form of the term of the original language;

ITEM 8 → No linguistic inconsistencies or conceptual errors were detected so no changes were performed;

ITEM 9 → No linguistic inconsistencies or conceptual errors were detected so no changes were performed;

ITEM 10 → No linguistic inconsistencies or conceptual errors were detected so no changes were performed;

ITEM 11 → No linguistic inconsistencies or conceptual errors were detected so no changes were performed;

ITEM 12 → the listening situation of announcements in the classroom did not happen in Italy as there are no loudspeakers for announcements in Italian schools. This item was replaced by the item 'how well do you understand the teacher in gym class?' and renamed as "listening in a gym class"

ITEM 13 → No linguistic inconsistencies or conceptual errors were detected so no changes were performed; "School play" was added to represent listening in a large room at elementary school

ITEM 14 → No linguistic inconsistencies or conceptual errors were detected so no changes were performed;

ITEM 15 → No linguistic inconsistencies or conceptual errors were detected so no changes were performed.
